# Perception of Plastic Surgery and the Role of Media Among Medical Students: Cross-Sectional Study

**DOI:** 10.2196/12999

**Published:** 2019-04-03

**Authors:** Hatan Hisham Mortada, Yara Aayed Alqahtani, Hadeel Zakaria Seraj, Wahbi Khalid Albishi, Hattan A Aljaaly

**Affiliations:** 1 Division of Plastic & Reconstructive Surgery, Department of Surgery Faculty of Medicine King Abdulaziz University Jeddah Saudi Arabia

**Keywords:** plastic surgery, perception, knowledge, medical students, media, King Abdulaziz University, Jeddah, Saudi Arabia

## Abstract

**Background:**

Although plastic surgery has been gaining a lot of popularity recently, there seems to be limited perception and a poor understanding of this field by both medical professionals, including medical students, and the general public. This might alter referral patterns as well as medical students’ choice to pursue a career in plastic surgery.

**Objective:**

The purpose of this study was to assess knowledge and perception of plastic surgery among medical students and to explore the influencing factors underlying particular beliefs.

**Methods:**

Data for this cross-sectional study were collected between August 22 and December 22, 2017. The questionnaire was formulated on the basis of our own study objectives and from available questionnaires with similar objectives. It was composed of 14 questions divided into three main parts: demographics, the specialty of plastic surgery, and media involvement and its effect on plastic surgery. The study was conducted via an online questionnaire among medical students in all years at King Abdulaziz University Hospital, Jeddah, Saudi Arabia. Data were considered significant at *P*<.05. All analyses were performed using SPSS, version 20.

**Results:**

A total of 886 medical students participated in this study. We achieved a response rate of 56.79%. The mean age of the participants was 21.2 years. The mean awareness score was 9.7 (SD 4.2) for female students and 8.3 (SD 4.2) for male students (*P*<.001). The condition most commonly known to be treated by a plastic surgeon was burns (70.3% of responses).

**Conclusions:**

Medical students do not have adequate awareness of plastic surgery, and early exposure to this specialty may enhance their awareness.

## Introduction

Plastic surgery is well defined as the specialty concerned with restoration, reconstruction, and enhancement of the function and appearance of body structures that are missing, defective, damaged, or misshaped. It includes both reconstructive and cosmetic surgery [1]. According to the American Society of Plastic Surgeons, nearly 17.1 million cosmetic procedures and 5.8 million reconstructive procedures were performed in 2016 alone; this represents an increase in cosmetic procedures of 132% since 2000 [2]. Despite this growth in the field, there seems to be limited perception and a poor understanding of this specialty by medical professionals, including medical students, and the general public [3].

Even though plastic and reconstructive surgeons require extensive surgical training and technical skills, they are mostly known for performing cosmetic surgeries [4]. An Indian study reported that plastic surgery is poorly understood in the medical community, as 12% of the participants thought that plastic and cosmetic surgeries were the same [3]. Similarly, a recent study conducted by Fraser et al [5] concluded that medical students have a skewed perception that is largely influenced by television. As these students go on to become practicing physicians, their misconceptions regarding plastic surgery may negatively affect the specialty by altering patient referral patterns and their decision to pursue a career in plastic surgery.

The number of Saudi plastic surgeons is relatively low in comparison to that in other parts of the world: Recent statistics showed the percentage of plastic surgeons in Saudi is 0.5% compared to 15.6% in the United States, 12.6% in Brazil, and 6.4% in China [6]. Therefore, this study aimed to measure the level of awareness about plastic surgery among medical students in Saudi Arabia in order to improve their perception and maybe their interest in plastic surgery as a choice in their specialty.

No data are available in the literature about medical students’ knowledge and perception of plastic surgery in Saudi Arabia. The purpose of this study was to assess knowledge and perception of plastic surgery among medical students in Saudi Arabia and to explore the influencing factors underlying particular beliefs.

## Methods

### Study Design and Data Collection

This cross-sectional study was conducted via an online questionnaire at King Abdulaziz University Hospital in Jeddah, Saudi Arabia. The survey was hosted freely on the Google survey webpage, and the link was sent by two randomly chosen representatives from each year (second to sixth year) who volunteered to distribute the questionnaire online via WhatsApp, wherein they had a master list of students’ names and their contact information. Participants were chosen via a multistage stratified random sampling method. Stratification considered gender and educational year (second to sixth). A total of 886 medical students participated in the study. The data were collected from August 22 to December 22, 2017. All participants were informed about the demands of the study, and those who agreed to participate were enrolled. Participants who refused to participate or failed to complete the questionnaires were excluded.

### Questionnaire Variables

The questionnaire was formulated on the basis of our own study objectives and from available questionnaires with similar objectives [7-9]. Both content and face validity were assessed by two experts. Internal consistency reliability was assessed using Cronbach alpha. The questionnaire was composed of 14 questions divided into three main parts: demographics, the specialty of plastic surgery, and media involvement and its effect on plastic surgery. The first part included age, gender, educational level, and academic grade point average (GPA). The second part aimed to assess medical students’ knowledge about plastic surgery. The total score for awareness was 21 and ranged between 1 and 21; a higher score indicated more awareness about plastic surgery. The final part involved questions aimed at determining the role of media in students’ perceptions of plastic surgery.

### Ethical Considerations

This study was approved by the Institutional Review Board and the Research Ethics Committee of King Abdulaziz University in Jeddah.

### Statistical Methods

Descriptive statistics were used for the baseline characteristics of all respondents, the frequencies and percentages of respondents who had chosen other specialties, the sources of information regarding plastic surgery, and the conditions treated by plastic surgery. The Student *t* test was used to compare the mean difference in awareness scores of all respondents according to different variables. A one-way analysis of variance test was used to compare the mean awareness score among participants across decisions regarding plastic surgery and the level of education. The Spearman correlation test was used to determine the correlation between the score achieved and age, educational level, and academic GPA. A Chi-square test generated *P* values according to different variables for participants who chose the plastic surgery specialty. Data were considered significant at *P*<.05. All analyses were performed using SPSS, version 20 (IBM Corp, Armonk, NY).

## Results

### Participants

A total of 886 medical students participated in the study, yielding a response rate of 56.79% (886/1560). The mean age of students was 21 years, and 50% were females. Students were from different levels of medical school—25.6% were in their final year—and 66% of the students had a GPA between 4.5 and 5 (Table 1).

**Figure 1:**
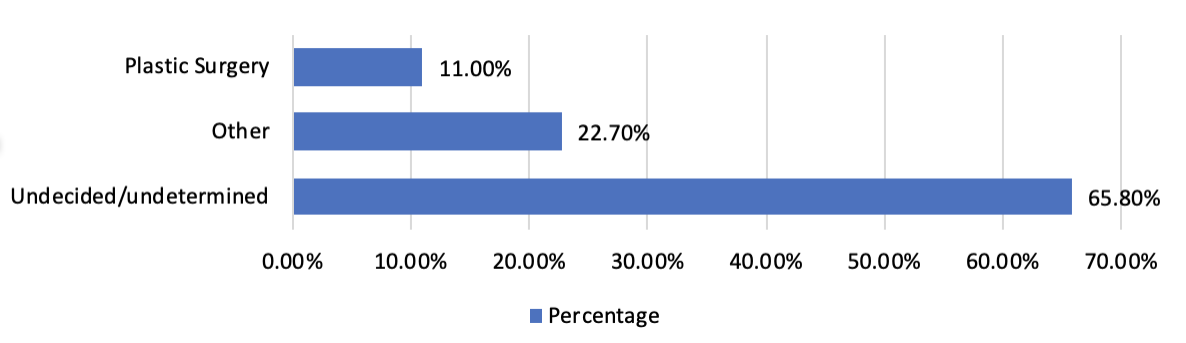
Specialty preferences among participants.

Among the sample, 65.8% of the students had not yet decided on their career specialty, 11% had chosen to pursue a career in plastic surgery, and 22.7% had chosen a different specialty (Figure 1).

### Knowledge of Plastic Surgery

The mean awareness score was 9.7 (SD 4.2) for female students and 8.3 (SD 4.2) for male students (*P*<.001). Those who had been exposed to a surgical discipline had a higher score (mean 10.4, SD 4.4) than those who had no exposure (mean 8.5, SD 4.1; *P*<.001). Students who had not decided on their career specialty had a lower score (mean 8.4, SD 4.1) than those who had chosen plastic surgery or any other specialty (*P*<.001). Sixth-year medical students had an awareness score of 10.5, which was higher than that of second-, third-, and fourth-year students (*P*<.001; Table 2).

**Table 1:** Summary of characteristics and responses of participants.

Characteristics and responses		n (%)
**Gender**		
	Female	440 (49.7)
	Male	446 (50.3)
**Educational level**		
	2nd year	156 (17.6)
	3rd year	220 (24.8)
	4th year	188 (21.2)
	5th year	95 (10.7)
	6th year	227 (25.6)
**Academic GPA^a^**		
	<2.5	1 (0.1)
	2.5-2.99	7 (0.8)
	3-3.49	29 (3.3)
	3.5-3.99	103 (11.6)
	4-4.49	252 (28.4)
	4.5-5	494 (55.8)
**Exposed to medically themed television**		
	No	325 (36.7)
	Yes	561 (63.3)
**Exposed to a surgical discipline**		
	No	648 (73.1)
	Yes	238 (26.9)
**Made a decision about choosing plastic surgery**		
	Yes	102 (11.5)
	No	39 (4.4)
	Other specialty	162 (18.3)
	Not decided	583 (65.8)

^a^GPA: grade point average.

**Table 2:** Achieved scores across various factors.

Factor		n	Mean score (SD)	P value
**Gender**				<.001^a^
	Female	440	9.73 (4.178)	
	Male	446	8.30 (4.196)	
**Exposed to medically themed television**				<.001^a^
	Yes	561	10.01 (4.029)	
	No	325	7.29 (4.060)	
**Exposed to a surgical discipline**				<.001^a^
	Yes	238	10.42 (4.296)	
	No	648	8.49 (4.110)	
**Wants to be a plastic surgeon**				.07^a^
	Yes	102	9.73 (4.446)	
	Other specialty or not determined	784	8.92 (4.213)	
**Made a decision regarding plastic surgery**				<.001^b^
	Yes	102	9.73 (4.446)	
	No	39	9.59 (4.278)	
	Other specialty	162	10.67 (3.984)	
	Not decided	583	8.39 (4.138)	
**Educational year**				<.001^b^
	2nd year	156	6.95 (4.011)	
	3rd year	220	8.66 (4.008)	
	4th year	188	8.84 (4.087)	
	5th year	95	10.01 (4.304)	
	6th year	227	10.49 (4.092)	

^a^By Student *t* test.

^b^By analysis of variance.

Burns were most commonly known to be treated by a plastic surgeon (70.3% of students), followed by rhinoplasty (67.6%) and breast reduction or enhancement (66.6%). The conditions least known to be treated by plastic surgeons were injuries to the nerves of the hands and legs (12.1% of students), tendon injuries of the hand (12.3%), and bedsores (13%) (Table 3).

### Role of Media in Perceptions

Of the students in the sample, 63.3% had been exposed to medically themed television programs. Their awareness scores were higher (mean 10, SD 4) than those of students who had not been exposed to these programs (mean 7.3, SD 4.1; *P*<.001; Table 1) The most commonly mentioned source of information was the internet (54.4%), followed by the television (44.7%; Table 4).

Spearman rank correlation was performed to test the correlation between the achieved score and age, academic year, and GPA (Table 5). There was a significant correlation between the score and age (*P*<.001; *r*=0.18). There was also a significant correlation between the score and academic year (*P*<.001; *r*=0.27), which indicates a weak positive correlation between the score and both age and academic year. There was no statistically significant correlation between the GPA and the achieved score (*P*=.45).

**Table 3:** Conditions treated in plastic surgery. Frequency refers to the number of students who were aware of the conditions treated in plastic surgery.

Condition	Frequency, n (%)
Burns	623 (70.3)
Rhinoplasty (nose job)	599 (67.6)
Breast reduction or enhancement surgeries	590 (66.6)
Botox	555 (62.6)
Cleft lip and palate (congenital)	536 (60.5)
Eyelid tears and cuts over the face	483 (54.5)
Congenital anomalies of ear and nose	460 (51.9)
Liposuction (fat aspiration)	456 (51.5)
Abdominoplasty (tummy tuck)	404 (45.6)
Fractures of the jaw and face	402 (45.4)
Hair transplantation	353 (39.8)
Sex-change surgery	352 (39.7)
Finger amputations	229 (25.8)
Diabetic foot wounds	153 (17.3)
Fractures of the hand	123 (13.9)
Bedsores	115 (13.0)
Tendon injuries of hand	109 (12.3)
Injuries to nerves of the hands and legs	107 (12.1)

**Table 4:** Sources of information regarding plastic surgery.

Source	Frequency, n (%)
Internet	482 (54.4)
Television	396 (44.7)
Friends	316 (35.7)
Snapchat	240 (27.1)
Instagram	234 (26.4)
Personal encounter	174 (19.6)
Twitter	127 (14.3)
Other	122 (13.8)
Teaching sessions	120 (13.5)
Magazines	87 (9.8)
Personal experience	86 (9.7)
Clinical rotations	81 (9.1)
Workplace	77 (8.7)
Facebook	26 (2.9)

**Table 5:** Spearman correlation test for the correlation between achieved score and age, educational level, and academic grade point average (N=886).

Variable	Correlation coefficient (*r)*	*P* value
Age	0.182	<.001
Educational level	0.267	<.001
Academic grade point average	0.025	.45

## Discussion

### Principal Findings

A total of 886 medical students participated in this study. The mean age of the participants was 21.2 years, and half of them were female. Almost 66% of the students had a GPA above 4.5/5. The mean awareness score was 8.3 (SD 4.2) for male students and 9.7 (SD 4.2) for female students (*P*<.001). The condition most commonly known to be treated by a plastic surgeon was burns (70.3% of responses), followed by rhinoplasty (67.6% of responses). As plastic surgery is a unique specialty that deals with everything from head to toe, it has no organ system of its own. Our data show that medical students lack a proper understanding of the specialty of plastic surgery. These findings are consistent with those of other studies that defined plastic surgeons as cosmetic surgeons only or did not recognize the surgeries that are commonly performed by plastic surgeons, such as hand surgery and cleft palate surgery [9,10]. Interestingly, these misconceptions are also held by other groups, including the public, primary care physicians, and residents [7,8].

Students in our study believed that plastic surgeons most commonly treat burns (70.3%), perform rhinoplasty (67.7%), and perform breast-reduction and enhancement surgeries (66.6%). In contrast, in a study from Pakistan, the perception was that plastic surgeons most commonly perform hair transplant surgery (89.9%), followed by facial scar surgery (88.0%) and rhinoplasty (83.4%) [11]. However, in a study performed in India, burns were the most frequently named condition (20.4%), but at a much lower percentage than that observed in our study (70.3%) [4]. Adeyemo et al [12] reported that the most commonly named procedures were liposuction (88.2%) and hair transplant surgery (84.4%); in contrast, liposuction was named by only 53% of our study participants. A similar study conducted in Pakistan among college students found that the internet was the main source of information about plastic surgery (88%) [11], which is in agreement with the results of our study (54.4%).

The revolution in various forms of media and social networking channels has made the conditions treated by plastic surgeons more recognizable. Medically themed series such as Grey’s Anatomy and House seem to be significantly associated with better awareness of plastic surgery among the students in our study. The internet and social media are considered a rich source of information for plastic surgery, as the majority of students identified them as their sources of information. Considering new trends in social media and the advertisements that serve to educate students about cosmetic surgery, the use of the internet and social media tools to promote a more accurate and realistic portrayal of medicine should be strongly advocated.

Medical students' perceptions about the different surgical disciplines may increase as they progress through their clinical years. Students in their final year in this study had significantly more knowledge about plastic surgery than their younger peers. Although medicine and surgery were considered essential in shaping the educational foundation of the students, their lack of knowledge about plastic surgery may have a negative impact on their chances of obtaining a residency in this field. Plastic surgery is an extremely competitive specialty, and it is mandatory for students who wish to pursue a residency position in this field to be involved in extracurricular activities that include electives and related research.

In our study, female medical students were more knowledgeable and aware of the discipline of plastic surgery than male students. This could be explained by the positive correlation between the awareness level and the GPA, as female students had a statistically significant higher GPA. In our study, we also found that 14.5% of male students wanted to pursue a career in plastic surgery compared to only 8.4% of female students, consistent with the findings of a study conducted in Riyadh, which showed that male medical students were more interested in plastic surgery as a specialty than female students [13]; this finding is in agreement with previous published articles in countries with cultural similarities such as Turkey and Jordan [14,15].

Specialty selection might vary according to gender, as shown in a study by Alshahrani et al: Family medicine was the most preferred specialty for women among Saudi medical students [16].

To our knowledge, this study is the first of its kind in Saudi Arabia to assess the perception and knowledge of medical students about plastic surgery. Many different modalities may improve awareness of this specialty. Self-explanatory brochures about this specialty and its different disciplines have been advocated as a tool to increase awareness. We recommend that future studies asses if there are any social or cultural components to the awareness score and analyze medical students’ interest of pursuing a carrier in plastic surgery.

### Limitations

Although our sample size was much larger than that reported in any other article in the field [4,5,11] and the study achieved its aim, there were some limitations that need to be highlighted. First, our study was cross-sectional, covered a short interval time, and was conducted at a single institution. In addition, there is a possibility that the responses and level of awareness are not representative of all medical students in Saudi Arabia. Second, response bias may have occurred because of the moderate overall response rate (56.79%), which was particularly affected by some students’ refusal to participate or failure to complete the questionnaire. This could be attributed to the lack of interest and time, which resulted in the exclusion of these students from the study.

### Conclusions

Medical students do not have adequate awareness of plastic surgery. Early exposure to this specialty may enhance their awareness. Internet and social media channels are rich sources of information and their use as educational tools should be encouraged.

## Abbreviations

GPA: grade point average
