# Metadata Correction: Perception of Plastic Surgery and the Role of Media Among Medical Students: Cross-Sectional Study

**DOI:** 10.2196/14352

**Published:** 2019-06-19

**Authors:** Hatan Hisham Mortada, Yara Aayed Alqahtani, Hadeel Zakaria Seraj, Wahbi Khalid Albishi, Hattan A Aljaaly

**Affiliations:** 1 Division of Plastic & Reconstructive Surgery, Department of Surgery Faculty of Medicine King Abdulaziz University Jeddah Saudi Arabia

The authors of “Perception of Plastic Surgery and the Role of Media Among Medical Students: Cross-Sectional Study” (Interact J Med Res 2019;8(2):e12999) wish to change the corresponding author from Hatan Hisham Mortada to Hattan A Aljaaly. The phone number and email address have been updated accordingly.

The correction will appear in the online version of the paper on the JMIR website on June 19, 2019, together with the publication of this correction notice. Because this was made after submission to PubMed, PubMed Central, and other full-text repositories, the corrected article also has been resubmitted to those repositories.

